# Sesquiterpene Lactones and Diterpenes: Promising Therapeutic Candidates for Infectious Diseases, Neoplasms and Other Chronic Disorders

**DOI:** 10.3390/molecules26051251

**Published:** 2021-02-26

**Authors:** Valeria P. Sülsen

**Affiliations:** 1 Instituto de Química y Metabolismo del Fármaco (IQUIMEFA), CONICET—Universidad de Buenos Aires, 1113 Buenos Aires, Argentina; vsulsen@ffyb.uba.ar; 2 Cátedra de Farmacognosia, Facultad de Farmacia y Bioquímica, Universidad de Buenos Aires, 1113 Buenos Aires, Argentina

Nature is an important source for the discovery of new bioactive compounds. Natural compounds constitute a strategic starting point for the development of novel drugs, since they exhibit a wide range of pharmacophores and a large number of chiral centers that allow the interaction with proteins and biological targets.

Terpenes are natural isoprene-derived compounds and are classified according to the number of carbon atoms in monoterpenes (C10), sesquiterpenes (C15), diterpenes (C20), triterpenes (C30), tetraterpenes (C40) and polyterpenes (C > 40). Many of these compounds are considered secondary metabolites in plants. They showed a variety of biological activities, such as anticancer, anti-inflammatory, antimicrobial, antiparasitic, antiviral, etc. They constitute the largest group of natural compounds with more than 50,000 molecules of diverse chemical structures. They are of great interest, both in the cosmetic and the food market as well as in the pharmaceutical industry [1].

Among terpenoids, sesquiterpene lactones and diterpenes stand out due to their role in human health and as a source of new drugs. The medicinal potential of plant terpenoids has been reviewed by Bergman et al. [2]. In this review, the sesquiterpene lactone artemisinin, isolated from the medicinal Chinese plant *Artemisia annua* (Asteraceae) is described. This compound and its semisynthetic derivatives are used nowadays for the treatment of malaria. Its mechanism of action is related to the heme metabolism of *Plasmodium* spp. Another sesquiterpene lactone mentioned by the authors is thapsigargin, a guaianolide-type sesquiterpene lactone produced by *Thapsia garganica* (Apiaceae), which has been proved to interact with pathways regulating Ca^2+^homeostasis in mammalian cells. The effect of the derivative mispsagargin is being evaluated in clinical trials for hepatocellular carcinoma. As regards diterpenoids, the antineoplastic agent paclitaxel isolated from *Taxus breviflolia* (Taxaceae) has been described. This compound acts as a microtubule stabilizer and a mitosis inhibitor. Paclitaxel is used for refractory ovarian cancer and metastatic breast cancer. It is currently obtained by semisynthesis from baccatins and from which docetaxel and analogs have been developed. Ingenol mebutate and prostratin are also diterpenoids with antitumor and anti-HIV activities. 

Other examples of bioactive terpenoids are forskolin, a labdan diterpene from *Coleus forskohlii* (Lamiaceae) and the sesquiterpene lactone arglabin, isolated from *Artemisia myriantha* (Asteraceae), as well as its derivative dimethylamino-arglabin, which have been demonstrated to be potential antitumor agents. Other sesquiterpene lactones with promising activity and which are under study are parthenolide and its analog dimethylamino-parthenolide, which are active against breast cancer cells, leukemia and pancreatic carcinoma cells; artemisinin, which has been studied for the treatment of different types of cancers such as breast and colorectal cancer [3,4]; and dehydrocostuslactone and costunolide for breast cancer and leukemia [5]. Other sesquiterpene lactones such as psilostachyin, psilostachyin C, helenalin, mexicanin, cumanin, deoxymikanolide, lychnopholide and goyazensolide were found to be active against tumor cell lines and against *Trypanosoma cruzi*, the causative agent of Chagas disease [6,7,8,9].

Over the last years, several updates have been published in *Molecules* regarding the potential of sesquiterpene lactones and diterpenes for the development of novel drugs. In this sense, Remy and Litaudon (2019) reviewed the properties of macrocyclic diterpenoids of plants of the Euphorbiaceae family and their capacity to inhibit chikungunya virus replication [10]. Jin et al. (2019), published a review on daphnane-type diterpenoids [11]. These authors highlighted the activities demonstrated for this type of compounds: anti-HIV, anticancer, antileukemic, neurotrophic, pesticidal and cytotoxic. Ullah et al. (2019) reviewed the pharmacological potential of steviol and isosteviol and derivatives as cytotoxic, antiviral, antibacterial, antihypertensive, anti-inflammatory and antihyperglycemic agents, among others [12]. Li et al. (2018) published a review describing *ent*-kaurane diterpenoids, specifically spirolactone-type diterpenoids, which exhibit attractive activities, especially antiproliferative activity [13]. Herrera Acevedo et al. [14] focused on the importance of in silico studies using sesquiterpene lactones for the detection of potential compounds for the treatment of leishmaniasis, schistosomiasis, Chagas disease and sleeping sickness. 

Apart from the reviews mentioned, research articles describing bioactive sesquiterpene lactones and diterpenoids have also been published in *Molecules*. A brief summary of selected articles published in 2019–2020 is also presented. 

The synthesis, crystallography and antileukemic activity of amino adducts of dehydroleucodine have been reported by Ordoñez et al. [15]. The cytotoxic activity of the sesquiterpene lactones was evaluated against acute myeloid leukemia cell lines. The proline adduct showed the highest antileukemic activity and was about 270 times more water soluble than the natural compound.

The isolation of the sesquiterpene lactones 4,15-iso-atriplicolide tiglate, methacrylate and isobutyrate from *Helianthus tuberosus* (Asteraceae) has been reported by Galkina et al. [16]. These compounds were evaluated against *Trypanosoma brucei rhodesiense*, *Trypanosoma cruzi*, *Leishmania donovani* and *Plasmodium falciparum*. The 4,15-iso-atriplicolide tiglate showed a promising activity and selectivity against *T. b. rhodesiense*, the etiologic agent of African human trypanosomiasis (IC_50_ = 0.015 ± 0.003 µM). Lenz et al. demonstrated that this sesquiterpene lactone inhibited trypanothione reductase (TR). This enzyme is responsible for the maintenance of the cellular redox state of the parasite [17]. Other analogs belonging to the furanoheliangolide-type sesquiterpene lactones were also found to inhibit TR.

The sesquiterpene lactones α-santonin, arglabin, schkuhrin II, vernolepin and eucannabinolide were loaded into polylactic acid (PLA) nanoparticles and evaluated against *T. b. rhodesiense.* Argablin, vernolepin and eucannabinolide showed trypanocidal activity with IC_50_ values of 3.67, 1.11 and 3.32 µM, respectively. None of the nanoparticle formulations were cytotoxic to mammalian cells [18].

The synthesis of oxygenated and oxy-nitrogenated derivatives of the sesquiterpene lactones cumanin, helenalin and hymenin was reported by Beer et al. [19]. The natural compounds and analogs were evaluated against human cancer cell lines. The silylated derivatives of helenalin were the most active (GI_50_ = 0.15–059 μM). The ditriazolyl cumanin was more active and selective than cumanin on the cell lines employed. This analog showed a GI_50_ of 2.3 µM and an SI of 227.9 on WiDr human colon tumor cell lines. 

Andrographolide is a labdene diterpene lactone studied by Li et al. (2020) [20]. The activity of three 14-aryloxy analogs of this diterpenoid was evaluated against Zika virus (ZIKV) and dengue virus (DENV). One of the derivatives (ZAD-1) showed higher activity against both ZIKV and DENV than the natural compound. The EC_50_ values against ZIKV and DENV-2 were 27.9 ± 1.7 µM and 22.6 ± 1.8 µM, respectively. 

The natural *ent*-kaurane diterpenoid adenanthin, isolated from *Isodon adenantha*, has shown activity against leukemic and hepatocellular carcinoma cells. This diterpene has been tested as a potential agent for the prevention of obesity [21]. Adenanthin inhibited adipogenesis in 3T3-L1 and mouse embryonic fibroblasts and reduced the growing body weight and adipose tissue mass during high-fat diet-induced obesity of mice.

Estafietin is a guaianolde-type sesquiterpene lactone isolated from *Stevia alpina* (Asteraceae). This natural compound and four semisynthetic derivatives were evaluated against *Trypanosoma cruzi* and *Leishmania braziliensis*. Epoxyestafietin was the most active compound against trypomastigotes and amastigotes, with IC_50_ values of 18.7 and 2.0 µg/mL, respectively. Regarding leishmanicidal activity, estafietin and 11βH,13-dihydroestafietin were the most active and selective compounds on *L. braziliensis* (IC_50_ values of 1.0 and 1.3 μg/mL, respectively) [19].

The in vitro and in vivo trypanocidal activity of eupatoriopicrin has been reported by Elso et al. (2020) [22]. This compound was active and selective against *Trypanosoma cruzi* amastigotes and tripomastigotes (IC_50_ = 2.3 µg/mL and 7.2 µg/mL, respectively). Eupatoriopicrin was also active in an in vivo model of Chagas disease, producing a significant reduction in the parasitemia levels in comparison with non-treated animals. Skeletal muscular tissues from eupatoriopicrin-treated mice displayed only focal and interstitial lymphocyte inflammatory infiltrates and small areas of necrosis. In contrast, infected mice treated with the vehicle showed severe lymphocyte inflammatory infiltrates with necrosis of the adjacent myocytes. 

The results detailed herein show the potential of sesquiterpene lactones and diterpenoids for drug discovery and development. The wide variety of skeletal types as well as the differences in oxidation and substitution patterns determine a wide range of biological activities, being the anticancer, antiparasitic, antiviral and the anti-inflammatory activities some of the most mentioned for these classes of phytochemicals.
